# Non-functioning pituitary macroadenoma manifesting as a cervical syndrome

**DOI:** 10.1016/j.clinsp.2024.100490

**Published:** 2024-09-13

**Authors:** Carla A. Scorza, Ana C. Fiorini, Josef Finsterer, Fulvio A. Scorza

**Affiliations:** a Disciplina de Neurociência, Universidade Federal de São Paulo/Escola Paulista de Medicina (UNIFESP/EPM), São Paulo, SP, Brasil; b Programa de Estudos Pós-Graduado em Fonoaudiologia, Pontifícia Universidade Católica de São Paulo (PUC-SP), São Paulo, SP, Brazil; c Departamento de Fonoaudiologia, Escola Paulista de Medicina/Universidade Federal de São Paulo (EPM/UNIFESP), São Paulo, SP, Brazil; d Neurology Department, Neurology & Neurophysiology Center, Vienna, Austria

## Correspondence

Sensory disturbances in the upper limbs as a manifestation of a pituitary adenoma are rare, usually due to concomitant carpal tunnel syndrome, and occur predominantly in Somatotropic Hormone (STH) ‒ producing tumors in pediatric patients with acromegaly.^1^^,^^2^ To our knowledge, a cervical syndrome with tingling and numbness in both upper limbs as the dominant manifestation of a non-functioning macroadenoma has not yet been described in an adult.

The patient is a 45-year-old woman who presented to the emergency room because she had been experiencing tingling in both upper limbs for ten days, followed by recurrent numbness in both thumbs and the tips of all other fingers for two days. She also complained of a mild, neck-like headache for about two weeks, which did not respond to the tizagelan prescribed by her GP. She also noticed a black bar or wandering black dot that had appeared in both fields of vision for two days.

Her medical history included a hysterectomy and bilateral adnexectomy four months earlier for uterine myxomatosis and benign ovarian cysts, a mild SARS-CoV-2 infection one year earlier, and recurrent, occasional transient double vision and bilateral temporal headaches for about six months, which she described as a pressure sensation that could not be triggered and did not require analgesic treatment. She denied acute or chronic stress in her professional or personal life. She did not take any medication regularly.

**Fig. 1:**
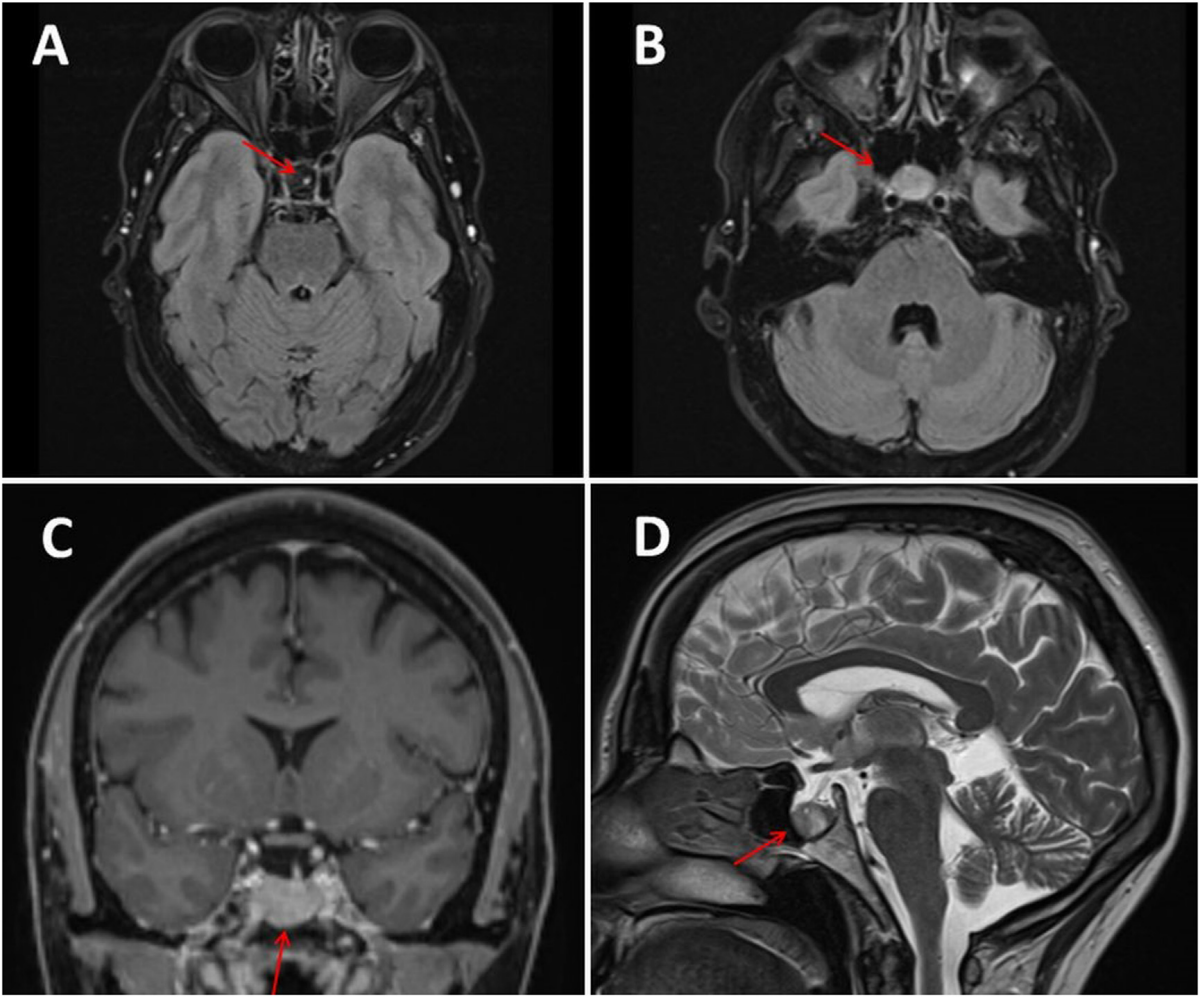
Cerebral MRI with contrast medium showing a mass lesion within the spheroid sinus and prominent pituitary stalk (panel A‒D), suggesting macradenoma of the anterior lobe of the pituitary gland.

Clinical neurological examination revealed muscle soreness in the neck, hyposmia (since SARS-CoV-2 infection), myopia, cold acras, mild acral cyanosis and only weak pulses in the upper and lower limbs. There was no clinical evidence of acromegaly, hypo- or hyperthyroidism, hypo- or hypercorticism, hypo- or hyperadrenalism, hypogonadism, or hyperprolactinemia. Cerebral MRI revealed a macroadenoma of the anterior pituitary (12 × 8 × 11 mm in size) and mild leukoencephalopathy (Fig. 1). The MRI of the cervical spine was normal. Blood tests showed slightly elevated lactate dehydrogenase, but STH, TSH, FSH, LH, and prolactin levels were normal. She also tested negative for SARS-CoV-2. Motor and sensory Nerve Conduction Studies (NCS) of the median, ulnar, and radial nerves were unremarkable. Ophthalmologic examination revealed no papilledema, retinopathy, or visual field defects. Doppler ultrasound of the arteries of the upper extremities was inconclusive. In addition to the macroadenoma, a cervical syndrome was diagnosed, and the patient was referred to the neurosurgeons for a possible hypophysectomy.

The presented patient is interesting because of cervical syndrome with tingling in both arms and numbness in all fingers for 10 days and neck headaches for about two weeks, as presentation of a non-functioning macroadenoma of the anterior pituitary. The fact that polyneuropathy, plexopathy, radiculopathy and carpal tunnel syndrome could be excluded as causal by means of NCS, normal cervical MRI, and that she also showed a slight coldness in the acres and a slight acral cyanosis speaks in favor of a causal relationship between the macroadenoma and the sensory disturbances. Differential causes of tingling and numbness that could alternatively explain these symptoms were ruled out. Adequate instrumental examinations revealed no evidence of polyneuropathy, carpal tunnel syndrome, plexopathy, radiculopathy, myelopathy, or myelitis. There was also no evidence of peripheral arterial disease in the aorta, subclavian, or upper limb arteries.

The pathophysiology of cervical syndrome is poorly understood.^3^ However, several hypotheses have been put forward to explain the underlying pathophysiology.^4^ The first hypothesis states that myofascial trigger points, i.e., specific areas usually located at the level of skeletal muscles that can trigger pain in specific regions of the body when pressure is applied, are involved in the pathophysiology of cervical syndrome.^4^ The pericranial muscles are the suspected trigger points for cervical syndrome. Excessive pericranial muscle contractions can lead to ischemia and the release of noxious agents such as substance P, which can trigger further pain.^4^ The second hypothesis is that autonomic dysfunction may play a role in the pathophysiology of cervical syndrome, particularly due to sleep disturbances.^5^ Sleep deprivation can lead to increased fatigue, which can result in the sympathetic nervous system overdrive, causing headaches.^5^ However, the patient did not report sleep deprivation. Sympathetic overstimulation can also lead to the contraction of the precapillary sphincters with consecutive acral hypoperfusion, resulting in coldness, cyanosis, and tingling in the acre. The impairment of the VNS could be due to an interruption of the connection between the pituitary gland and the hypothalamus due to a deformation of the pituitary stalk as a complication of the macroadenoma. The third hypothesis is that Nitric Oxide (NO) and its prodrug Glyceryl Trinitrate (GTN) cause headaches by stimulating nociceptors, which is why inhibiting NO production or blocking steps in the NO-cGMP signaling pathway or scavenging NO may reduce headaches.^6^

In summary, a non-functioning pituitary macroadenoma in a patient without acute or chronic stress or sleep deprivation may manifest with cervical syndrome manifested by sensory disturbances in the upper extremities.

## Authors’ contributions

JF was responsible for the design and conception, discussed available data with coauthors, wrote the first draft, and gave final approval. CS, AF, and FS: Contributed to literature search, discussion, correction, and final approval.

## Availability of data and material

All data are available from the corresponding author.

## Ethical approval

Not applicable.

## Consent to participation

Not applicable.

## Consent for publication

Not applicable.

## Declaration of competing interest

The authors declare that the research was conducted in the absence of any commercial or financial relationships that could be construed as a potential conflict of interest.
